# The care.data consensus? A qualitative analysis of opinions expressed on Twitter

**DOI:** 10.1186/s12889-015-2180-9

**Published:** 2015-09-02

**Authors:** Rebecca Hays, Gavin Daker-White

**Affiliations:** NIHR Greater Manchester Primary Care Patient Safety Translational Research Centre (PSTRC), Manchester Academic Health Science Centre, University of Manchester, Williamson Building, Oxford Road, Manchester, UK

## Abstract

**Background:**

Large, integrated datasets can be used to improve the identification and management of health conditions. However, big data initiatives are controversial because of risks to privacy. In 2014, NHS England launched a public awareness campaign about the care.data project, whereby data from patients’ medical records would be regularly uploaded to a central database. Details of the project sparked intense debate across a number of platforms, including social media sites such as Twitter. Twitter is increasingly being used to educate and inform patients and care providers, and as a source of data for health services research. The aim of the study was to identify and describe the range of opinions expressed about care.data on Twitter for the period during which a delay to this project was announced, and provide insight into the strengths and flaws of the project.

**Methods:**

Tweets with the hashtag #caredata were collected using the NCapture tool for NVivo. Methods of qualitative data analysis were used to identify emerging themes. Tweets were coded and analysed in-depth within and across themes.

**Results:**

The dataset consisted of 9895 tweets, captured over 18 days during February and March 2014. Retweets (6118, 62 %) and spam (240, 2 %) were excluded. The remaining 3537 tweets were posted by 904 contributors, and coded into one or more of 50 sub-themes, which were organised into 9 key themes. These were: informed consent and the default ‘opt-in’, trust, privacy and data security, involvement of private companies, legal issues and GPs’ concerns, communication failure and confusion about care.data, delayed implementation, patient-centeredness, and potential of care.data and the ideal model of implementation.

**Conclusions:**

Various concerns were raised about care.data that appeared to be shared by those both for and against the project. Qualitatively analysing tweets enabled us to identify a range of concerns about care.data and how these might be overcome, for example, by increasing the involvement of stakeholders and those with expert knowledge. Our findings also highlight the risks of not considering public opinion, such as the potential for patient safety failures resulting from a lack of trust in the healthcare system. However, caution is advised if using Twitter as a stand-alone data source, as contributors may lie more heavily on one side of a debate than another. A mixed-methods approach would have enabled us to complement this data with a more representative overview.

## Background

In recent years, technological improvements across the globe have led to massive increases in the amount of data that can be collected, stored, and processed. Large and complex collections of data, or ‘big data’, can be used to benefit populations by addressing issues of national concern [1]. In healthcare, integrated systems and datasets can be used to identify risk factors for health conditions, and opportunities for their prevention and management [2]. Big data is seen as having the potential to revolutionise healthcare, by enabling the identification of problems and treatments faster than would otherwise be possible, and more personalised and accurate predictions of risk [3]. However, there are downsides to such large, integrated datasets, such as the risk of individual identification and loss of privacy, and the impracticality of seeking informed consent from each patient before accessing their data [4]. As a result, big data initiatives such as the care.data project proposed by NHS England, are often controversial.

### Care.data

At the beginning of 2014, NHS England announced they were spending £1 million on delivering a leaflet entitled “Better information means better care” to 22 million homes [5]. The leaflet formed the first part of a publicity campaign in relation to an initiative known as “care.data,” whereby aspects of patients’ primary care medical records would be automatically uploaded monthly to a central database [6]. However, the leaflet did not contain the words “care.data” or an opt-out form. Rather, readers were instructed: “If you have any questions or are not happy for information about you to be shared, speak to your GP practice.” The leaflet also contained links to a dedicated telephone helpline and the web sites of NHS Choices and The Health and Social Care Information Centre (HSCIC) [7].

Care.data led to intense debate that included editorials and correspondence in high-ranking scientific and medical journals, articles in news media, and activity on social media sites. Various organisations also conducted surveys to identify how many people had knowingly received the “Better information means better care” leaflet, felt they understood what care.data was, supported or opposed the project, and intended to opt out [8–10].

As practitioners of patient safety research, the authors took a keen interest in the issues raised and the potential implications for healthcare. They began following the development of the project, especially through the commentary on Twitter where users were discussing the issue in-depth. On 19 February 2014, Tim Kelsey, the National Director for Patients and Information at NHS England published a statement announcing that uploads of data from general practices would be put back to Autumn 2014 from the planned April start date [11]. Following this announcement, news and social media activity in relation to care.data increased.

### Social media

Social media are “web sites and applications which enable users to create and share content or to participate in social networking” [12]. The two most popular social media sites among UK adults aged 16+ are Facebook and Twitter [13]. Communication is the primary reason for using both sites but they provide different opportunities for interaction and are used for slightly different purposes. Whilst Facebook is used to share opinions and photographs with friends, Twitter is used as a news source and to keep up with current events [13, 14].

### Twitter

On Twitter, users can post messages or micro-blogs, called ‘tweets’, of up to 140 characters. Tweets can include links to other content (pictures or videos) and websites (e.g. articles from digital editions of newspapers). Approximately 500 million tweets are sent per day and Twitter has 316 million active users [15]. Twitter users’ accounts enable them to follow other users, subscribe to their tweets, and be followed. Most tweets are public and can be viewed by anyone, with or without a Twitter account, but some users protect their tweets so they are only visible to approved followers.

As a social environment, Twitter has its own etiquette or code of good practice. This includes the use of hashtags, which are words, acronyms, or phrases starting with the ‘#’ symbol and continuing without spaces. Hashtags enable users to search for information, and follow and contribute to discussions on particular topics. Users can repost other users’ tweets, sharing them with their followers by using the retweet function or manually copying and reposting, adding the letters RT to the beginning. Twitter users can also engage in conversations by including each other’s usernames, which start with the ‘@’ symbol, in tweets. However, unsolicited and unwanted tweets are considered spam and violate Twitter rules.

Within the healthcare sector, Twitter can educate patients and promote positive health behaviours such as smoking cessation and immunisation, and improve healthcare by informing providers about the latest research and guidance [16]. Healthcare researchers are also using public tweets as a source of data. The number of tweets including specific keywords can be counted to monitor current trends in health-related behaviours and infectious disease, and the sentiment of tweets can be assessed and counted to evaluate healthcare services or to review public opinion on planned service changes, such as the passing of the Health and Social Care Bill in England [17, 18]. Alongside these quantitative methods, some studies have carried out qualitative content analysis of a random subsample of approximately 1000 tweets. One such study regarding care quality in NHS hospitals in England, concluded healthcare services should not be judged on the basis of tweets alone but suggested the content of tweets could provide new insights about the views of the public (complementing other data sources), and identify where improvements may be needed [19].

The aim of the study described in this article was to identify and describe the range of opinions expressed about the care.data project on Twitter for the period during which the delay to this project was announced, analysing the data in such a way as to provide insight into the strengths and flaws of the project. Exploring the nature and character of the Twitter discourse in-depth enabled us to provide an overview of the concerns people had about care.data and its implementation, and the reasons why they supported the project or the goals of its implementation, highlighting differences of opinion and identifying where there appeared to be a consensus. This objective was realised using methods of qualitative data analysis normally employed in studies of texts transcribed from focus groups, ethnographic observations (e.g. field notes) or semi-structured ‘depth’ interviews.

## Methods

NCapture is a web browser extension for NVivo 10 that can be used to create a chronological dataset or ‘batch’ of tweets, working backwards from the time of the ‘capture’. The number of tweets it is possible to capture is determined by Twitter and the amount of traffic or data flow on the site at that time [20]. Therefore, we sought to collect data at set intervals until we had gathered a target of approximately 10,000 tweets.

We logged into Twitter (as @Multimorbidity) and searched for all tweets containing the hashtag #caredata. The NCapture tool (for Internet Explorer) was used to convert the search results into a dataset. Initial tests with this tool suggested we could collate an almost continuous dataset by carrying out captures at a similar time, every three working days (from Monday-Friday). Therefore, the capture was repeated several times around 5 pm on each assigned day, and the capture with the largest number of tweets (that is, the one covering the longest time period) was retained. The target number of tweets was reached on the fifth day. This final capture encompassed all the tweets captured on the fourth day so the latter was deleted, and four batches of tweets were imported into NVivo.

The dataset was checked to ensure it only contained public tweets. To make the identification of authors and those mentioned more difficult, quotes are presented anonymously and the names of individuals and organisations (except for general references to the NHS) have been replaced with their profession or type (where known). Links to other content have also been replaced with the word ‘link’. The study was discussed with the University of Manchester Research Ethics Committee who confirmed it would not require ethical approval. However, we recognise there is ongoing debate about the ethical issues raised by the use of this type of data for research purposes [21].

### Data analysis

We began by exploring the automated features of NVivo to generate and count key words as contained in tweets. However, this was not found to be a useful way of exploring the data. Instead, we used established methods of qualitative data analysis, as normally used in semi-structured interview studies. Following grounded theory methods of data analysis, the tweets were read in sequential order, line by line, and coded in iterative fashion according to an evolving list of themes [22, 23]. In this manner, we were able to familiarise ourselves with the Twitter discourse and characterise the basic thrust of each tweet in a more nuanced way than would be possible to garner by just reading through the search results for #caredata on Twitter or *via* automated methods. Retweets and spam tweets were excluded manually.

The second author initially coded all of the included tweets. The codes were reviewed and revised by the first author, who combined and organised these into a thematic framework. Themes were sub-divided into two equal subsets, with each author conducting a more detailed evaluation of the codes in their set and producing a summary of each key theme. These summaries were reviewed and amended by both authors until a consensus was reached. The identity of the authors of tweets was not taken into account during the analysis. The overall aim of the analysis was to characterise the main issues articulated in the Twitter discourse concerning care.data.

## Results

### Overview

**Fig. 1:**
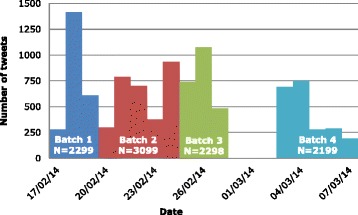
When and how many tweets were captured

The dataset consisted of four batches of tweets captured on 19, 24 and 27 February, and 7 March 2014. It contained 9895 tweets, which were posted over 18 days from 5.35 pm on 17 February to 4.59 pm on 7 March (see Fig. 1). However, there were gaps between the batches of 20, 27 and 92 h respectively so we did not capture any of the tweets posted during a third of this time (138 of 431 h, 31.9 %).

A total of 6118 (61.8 %) were retweets and 240 (2.4 %) were identified as spam and excluded from the analysis. The 3537 ‘original’ tweets were posted by 904 contributors. The top 5 contributors posted over a fifth of the tweets (756, 21.4 %), with one person posting 307 (8.7 %). Each user contributed an average of 4 tweets but the vast majority (743, 82.2 %) posted less than this. The discussion involved employees and senior managers from Non-Departmental Public Bodies (NDPBs), primary care workers such as General Practitioners (GPs), Members of Parliament (MPs), academics (e.g. from medicine, health or IT security), journalists, privacy campaigners, patient advocacy groups, activists and members of the public.

There was considerable confusion (seemingly from all perspectives) about what care.data would involve; that is, what data would be available to whom in what form. Tweets seemed to reflect a variety of strategic positions including: advancing ‘pro’ and ‘anti’ care.data arguments, encouraging people to opt out or not, providing links to information, correcting the perceived errors of journalists and politicians, and calling for the project to be abandoned or changed. There was also illiberal use of humour and sometimes personal attacks on staff from NDPBs, journalists, MPs and others.

The Twitter discourse encompassed a wide range of topics and addressed a series of related issues. Tweets were coded into one or more of 50 sub-themes, which were organised into 9 key themes (see Table 1). Eight of these highlighted the concerns people had about the care.data project or its implementation, and included tweets from users who were both against and broadly supportive of care.data, whereas the final theme pertained to the potential of care.data and the ideal model of implementation. These findings are considered in further detail below.

**Table 1:** List of key themes and sub-themes

Key themes		Sub-themes	
1	Informed consent and the default ‘opt-in’	1	Opt-out versus opt-in
		2	Informed consent
		3	Patient access to GP records
		4	Apathy regarding default ‘opt-in’
2	Trust	5	Lack of trust
3	Privacy and data security	6	Identifiable data
		7	Privacy
		8	Security issues
		9	Confidentiality of patient data
		10	care.data has risks
4	Involvement of private companies	11	Data accessed by insurance companies
		12	Data for sale
		13	Concerns about the involvement of a private IT company
		14	Data going to other private companies
		15	Care.data linked to NHS privatisation
5	Legal issues and GPs’ concerns	16	Data protection
		17	Legal issues
		18	GPs' concerns
		19	Concerns about adding genomic data later
		20	Police access to care.data
6	Communication failure and confusion about care.data	21	Communication failure
		22	Confusion about what care.data is
		23	care.data as a media event
		24	care.data leaflet and junk mail
		25	Conflicts of interest
		26	Public ignorance about care.data
		27	Mainstream media reports on care.data
		28	PR and presentation of facts
		29	Critics accused of scaremongering
7	Delayed implementation	30	Delay to implementation
		31	Role of activists or activism
		32	Flawed project
		33	Change management
		34	Another NHS fiasco
		35	Pause will not affect implementation
		36	A failed brand
		37	Costs of care.data project
		38	Technological problems
		39	Accuracy of medical records
8	Patient-centeredness	40	Arrogance
		41	Lack of patient and public involvement
		42	Lack of patient-centeredness
		43	Patient ownership of medical data
		44	Lack of engagement
9	Potential of care.data and the ideal model of implementation	45	Pro care.data viewpoint
		46	Ideal model of implementation
		47	Benefits of open data
		48	Accusing critics of scaremongering
		49	Use of patient data is not new
		50	Moral imperative for care.data

As is conventional in published qualitative work, we have characterised the frequency of tweets using descriptors such as “often” and “some”, and presented examples of tweets under each sub-heading to illustrate some of the opinions expressed [24–27]. These characterisations should not be considered accurate estimates of frequency as no formal quantitative analysis has been carried out, and it should also be noted that the selected quotes do not necessarily represent the general or majority opinion.

### Informed consent and the default ‘opt-in’

Tweets, including some that referred to surveys, suggested that most patients were not just inadequately informed about care.data and their right to opt out but were not aware of the project. Some people reported that GPs were ultimately responsible for informing patients about care.data but this policy was questioned in light of the already high workloads in primary care. Tweets also reflected the variations in different GPs’ ways of informing their patients (or not).

*Doctors should be helping patients understand implications of #CareData @[medical body] How much consultn time is this taking? [link]*

Some pointed out that the information belongs to the patients. That it is their data (ownership) and they should be able to choose who sees what information about them, and who doesn’t. However, it was also highlighted that patients struggle to access their own healthcare records and that this should be addressed before patients can make an informed decision about sharing this information.

*Seemingly radical idea: let PATIENTS control who can access their personal medical data! #caredata*

Twitter users argued that the automatic opt-in (with the option to opt out) was not equivalent to consent but a form of presumed consent and unethical, especially in light of patients’ lack of awareness. The issue of consent was a key concern for those that supported the idea of care.data but did not agree with the way it was being introduced. Users tweeted to inform people how they could opt out, or to say they had opted out and encourage or even instruct others to do the same. Concerns were also expressed that information would not be removed from the database if a person opted out after some of their data had already been transferred.

*Very conflicted re: #caredata - want to support anything that aids healthcare research, but can't see how 'opt out' option is valid consent*

*@[lawyer] @[lawyer] I've opted out of #caredata you all should before its too late*

### Trust

Tweets often disclosed a lack of trust surrounding the care.data project, and the government and NHS in general. This appeared to arise from a perceived lack of transparency, which some interpreted as dishonesty or incompetence. Opting out was seen as a way of declaring this lack of trust. Those in support of the idea of care.data called on relevant parties to establish trust to stop people from opting out, or suggested people opt out now but opt in once trust has been gained. However, the project was seen as having an “up hill climb” ahead, following “a shambles of a start”.

*Don't trust UK governments to protect YOUR personal data (& why would you)? —send them a message:Opt-out of #caredata.*

*#caredata @[NDPB] @[NDPB staff] @[NDPB staff] you will know when you have earned the nation's trust when informed people begin opting back in.*

The third batch of tweets included a number of references (85, 3.7 %) to the involvement of a private IT company in the care.data project, following a report in the Daily Telegraph that they had “been given the contract to extract patient records from GP surgeries” [28]. Users expressed shock and questioned the reasoning of the decision makers in awarding “a big juicy contract” to a company that had received criticism regarding its handling of another government contract. Public trust appeared to diminish further at this point and the number of people opting out was expected to increase.

*This government refuses to learn lessons: #[private company] are now responsible for our medical records. Terrifying. #caredata*

*Considering [private company]'s infamous reputation, does that just mean the whole country's about to opt out? #CareData*

### Privacy and data security

Tweets expressed concerns about the confidentiality of patients’ medical information, focusing on privacy rights and the security of the data collected. In the worst case scenario, it was suggested that fears over confidentiality would stop people from seeking healthcare, especially those who already find services difficult to approach, such as those with mental health problems.

*I've always struggled to open up about M/H to GP - These days I worry where anything I disclose will end up! o_O #[private company] #caredata #mhealth*

Privacy concerns were heightened due to lack of clarity regarding what data will be anonymised or pseudo-anonymised, at what stage and by whom. Tweets explained the differences between anonymisation and pseudo-anonymisation and expressed concerns that General Practices do not have the capacity or capability to do this. Users were also concerned that it would be easy to carry out ‘jigsaw identification’ from the pseudo-anonymised records, and re-identify individuals. There was also concern that the public lacked awareness and understanding of data security, the difference between anonymisation and pseudo-anonymisation, and risks of re-identification of individuals.

*#NHS #caredata Too many, esp older, folk aren't data/internet savvy enough to understand the implications. #dataprotection #privacy #bigdata*

Security concerns included the possibility that data could be leaked or lost when being transferred, and that the care.data database could be hacked. On occasion, it appeared as though a lack of trust in care.data had been carried over from other government or private sector IT “failures” or breaches of data security. Contributors requested more information about the steps that would be taken to ensure data security, and “stiff penalties” for those that infringe “medical privacy”. Yet some saw care.data as a trade-off between healthcare and privacy.

*@[private company] much like all massive IT projects (I'm thinking banks). They never turn out well. #caredata*

*@[MP] I'd be delighted to read what the "package of #caredata safeguards" contains. Are they documented somewhere please?*

*The #caredata debate sounds like security (of your life via better healthcare) vs. liberty (privacy of medical data) - familiar to US ears*

### Involvement of private companies

Many expressed concerns about who their data would be made available to, and there was much negativity at the suggestion this could be ‘sold’ to insurance and other private companies. Users suspected private companies with access to care.data would carry out illegal re-identification of individuals, and use this information to determine who they offer financial services (such as mortgages) to. Users also connected these issues with the privatisation of healthcare services and accused the government of “trying to make NHS into a for-profit organisation” and “losing sight” of its core goals.

*If NHS data is sold to insurers, & NHS continues to privatise, are those in ‘worse health’ (i.e. poor) areas likely lose out? #CareData*

These issues were mostly discussed in the second batch of tweets but tweets on the sub-theme ‘data going to other private companies’ were prevalent in the final batch after revelations early in March 2014 that Hospital Episode Statistics (HES) were being used by marketing companies. For some, the involvement of private companies out-weighed the potential benefits of care.data.

*Concerns have been raised that hospital records are being used by private firms to advise companies how to target their marketing #caredata*

*I don't care how useful the data would be to the NHS if data is also being handed to private companies to do with as they please. #caredata*

### Legal issues and GPs’ concerns

It was questioned whether or not legal or data protection experts had been involved in the development of care.data. Others argued that care.data is exempted from the provisions of the Data Protection Act (DPA) under the Health and Social Care Act (2012) which was interpreted as giving the Secretary of Health unilateral powers to release patients’ medical information. Whilst this study was not concerned with the factual accuracy of such statements, this debate again highlights the confusion or lack of clarity surrounding the care.data project.

*[link] - care.data opt-out has no legal force, so can be ignored completely. Data is DPA exempted. #ico #caredata #NHS #DPA*

Despite containing the #caredata hashtag, a substantial proportion of the tweets about medical data protection related to media reports concerning HES. This further highlights the confusion around care.data and suggests there is a tendency for it to be mixed-up with other initiatives. However, it was also evident that some sections of the media or Twitter users could have deliberately overlooked such distinctions in an attempt to soil the care.data brand using a form of guilt by association.

There was also a debate about whether GPs would be liable for breaches in patient confidentiality. This was tied into concerns that people had not been adequately informed about care.data. Results of surveys were referred to in tweets, suggesting that many GPs believed their patients were not aware of care.data, and a substantial minority were planning to opt out of care.data themselves. There was also consternation among some GPs that professional medical bodies had approved the scheme in its present form.

*@[journalist] you should also note that as things stand if just one pt claims not aware #caredata the GP is liable for DPA breach*

*Three quarters of GPs do not think patients have been sufficiently informed about #caredata [link]*

*Over 40 % of GPs intend to opt themselves out of #CareData scheme [link]*

### Communication failure and confusion about care.data

No matter what people’s views were on other aspects of care.data, there was almost universal agreement that aspects of the publicity and communication campaign were seriously flawed. A specific communication issue related to the leaflet sent to households in an attempt to inform the public about care.data. Tweets suggested that many households either did not receive the leaflet or did not notice it as a result of, what one user termed, the “junk mail fiasco”. At a Data Protection Practitioner Conference on 3 March 2014, a show of hands revealed approximately half of Data Protection Officers had not received the leaflet, including the Information Commissioner. In addition, those arguably “most concerned with privacy”, who had opted out of ‘Royal Mail Door to Door’, did not receive the leaflet, as doing so stops Royal Mail from delivering unaddressed items. For those who had not received the leaflet, some people tweeted a link to where it could be found online. However, not having received a leaflet was not always seen as a bad thing given the poor quality of the content. Some saw the leaflet as a waste of money, and a one-off mailout as an insufficient way to inform the public about care.data.

*@[unknown] apparently everyone got one - but it looked like just another piece of junk mail [link] #caredata #NHS*

*@[unknown] Indeed. I never even received the @[NDPB]'s misinformation sheet about #caredata*

*The leaflet sent for #caredata is a great example of fail-by-misplaced-moneysaving.*

The media were also seen as perpetrating an ill-informed debate, and some of those speaking about the subject, including politicians, journalists and twitter users, were accused of conflicts of interest and factually incorrect statements. Two commonly mistaken ‘facts’ repeatedly pointed to were the assertions that care.data involved “anonymous” collection and dissemination of patient medical records, and the assumption that the purpose of this sharing of primary care data was in order to marry up records and information for the purposes of individual patient care. The considerable confusion around the purpose and anonymity of care.data, as articulated by politicians and journalists commenting on the subject, seemingly exacerbated the communication failures highlighted above. Extreme examples in this regard involved repeated ridiculing of spokespeople from NDPBs referring to care.data as “anonymous” in national media (e.g. broadcast BBC interviews); a report in *Pulse Today* that staff from NDPBs had been criticised for posting inaccurate tweets about care.data; and the case of an MP who had apologised to the Speaker for making inaccurate statements about care.data in the House of Commons.

*Excellent bit of blogging here from @[GP] on #caredata & data literacy amongst journalists [link]*

*@[GP] @[GP] most pts unaware re #caredata if are aware think it's about data starring [sharing] between clinicians*

*#caredata rollout delayed by 6 months; plenty of time for [NDPB] to admit this is not data sharing for direct care but for secondary use*

Some critics of the initiative were portrayed as “scaremongering” whilst those speaking in favour of care.data were labelled as “spin doctors”. Inaccurate and ill-informed statements on the part of apparently uncritical supporters of the care.data project led to accusations that NDPBs and others were involved in “spinning” care.data rather than putting forward facts and issues in an open and transparent manner. However, some proponents felt that the benefits of care.data had not been discussed fairly in the media.

*@[GP] concerns are one thing, but the benefit side of the equation needs to be aired. #caredata*

Overall, the publicity campaign (including the leaflet, and radio and TV interviews) was seen as insubstantial, partial, light on detail and characterised by contradictory statements. In extremis, those with responsibility for informing the public were accused of incompetence.

*#NHSPatientdata scheme handling a 'masterclass in incompetence' #CareData #NHS [link] [link]*

*@[medical charity] People are now wanting to opt out of the SCR [Summary Care Record] due to confusion & concerns over care.data #caredata*

On the face of it, the wide acknowledgments concerning the flaws in the publicity campaign appear as at least one of the reasons why the care.data initiative was put back. However, critics complained that a pause to “explain the benefits” was still insufficient or that care.data is so fundamentally flawed that improving communicative aspects does not go nearly far enough.

*#caredata So they think it's all about explaining better. Words lipstick and pig come to mind; still a pig, no matter how expertly applied.*

### Delayed implementation

Given the time period during which tweets were collected, it was perhaps unsurprising that the delay was a popular topic for discussion. A range of sub-themes related to delayed implementation including ‘technological problems’ and ‘pause will not affect implementation’. It was also associated with ‘role of activists or activism’ in that some people interpreted the delay as a victory for the social media information campaign. Others used the announcement of the delay as evidence of the “flawed” nature of the care.data project in support of the furtherance of what appeared as an opt-out campaign by some activists.

*Wow I have just heard news #caredata to be delayed for 6 months.Congratulations to all tweets who keep this campaign going #patientconsent*

*All those local #[patient advocacy group] who have raised their concerns about #caredata Check out the story @ [link] for more info!*

*Good to see #caredata rollout delayed. Potentially beneficial #opendata possibilities, but lack of public awareness of opt-out very worrying*

### Patient-centeredness

Overall, the care.data project was considered to have adopted a paternalistic or “doctor knows best” approach. The project leaders and decision makers were seen as arrogant and patronising, and were also accused of lying about care.data; perhaps under the misguided assumption that this was in the patients’ best interests, with one user describing the project as an “arrogant incompetent ideology driven hubristic mess”. In extreme cases, care.data was articulated as an overt attempt to deceive the public or even representing a pursuit of “ideology over patient interest”.

*@[journalist] #caredata campaign redolent of past age in which doctors tell well-intentioned lies to patients, for their own good of course.*

*This whole #caredata thing with [NDPB] is a mess: how difficult is it to release a clear, unambiguous & truthful statement?*

The lack of patient-centeredness was emphasised by the lack of patient and public involvement in the development of care.data. This was seen as a key reason why issues of public concern had not been taken into account. Whilst some valued the project’s delay as an opportunity for people to get informed about care.data, there were concerns that the additional time would only be used to inform patients rather than listen to them and respond to their concerns by modifying the project. In the extreme, the project leaders were considered to have “bunker mentality, disdain for public opinion, [and] patronising arrogance”.

*@[NDPB] continue to treat us like children. The #caredata pause is to "explain its benefits". Well, it's easier than having a dialogue.*

*@[journalist] Businessmen are now in charge at the top. That's the nub of the problem. They don't even grasp our concern. #caredata*

Connections were made between the lack of patient involvement in the care.data project and patients’ lack of access to and control of their health records in general. Some stressed the potential importance of patient involvement in the management of medical records, and their ability to perform ‘quality control’ checks on the information. There was a perception that improving the quality of patient healthcare records should take priority over care.data.

*@[healthcare website] If we the patients could record our own symptoms & this was collected by #caredata Just think how that would change health*

### Potential of care.data and the ideal model of implementation

A small number of people tweeted that they had absolutely no concerns about care.data. Contributors pointed to existing uses of patient data and contexts in which large numbers of people are happy to share other personal information, for example, through online banking, shopping loyalty cards and social media. However, the main argument for care.data was that the benefits of such data sharing (for populations) outweigh any risks (for individuals).

*Most are happy to share financial data online but not healthcare data? #caredata*

*Disappointed #caredata is delayed. We can't all denote [donate] a kidney but we can denote [donate] our data to improve health of next generation. #opendata*

Tweets that were broadly supportive of care.data appeared to be far fewer in number than those that were critical. However, it would be simplistic to suggest people were either ‘for’ or ‘against’ the initiative. Those who acknowledged the potential benefits of care.data appeared disappointed by how the project had been carried out to-date, and some worried that too much damage had been done and the project could not be saved, especially if the delay was not used constructively. A sizeable proportion of contributors appeared as ‘critical friends’, including an ‘expert minority’ who were engaged in jointly working out what care.data actually involved and what could be done to improve the perceived faults.

@[IT developer] What bits do you disagree with? I'd be in favour of #opensource #caredata, but am radically against *this* implementation.

Contributors on all sides of the debate made a discernible attempt to suggest ways in which care.data could be made to work in ethically and technologically superior ways. Twitter users called for the project to be opt-in, with the right to withdraw at any time, or to have a simplified opt-out process, for example, by making it possible to do this online. The ideal model of implementation was said to require increased transparency, public awareness and involvement, and data security. Some contributors suggested the data should be anonymised, whereas others called for specific laws to protect privacy and clear guidelines regarding who has access to this data. Others wanted more options and the ability to choose who their data could be accessed by or for what purpose. One user suggested there should be “transparent trials of #caredata implementation with risk/benefit analysis”. However, the key message was that the delay to the project should be used to fully inform the public, listen to their views and reconsider how the project was to be carried out.

*Need an independent panel of patients, GPs, lawyers, ethicists, researchers and commissioners to decide who should have access to #caredata*

*@[MP] #caredata needs a complete rethink - proper anonymisation, clearer information and more honesty. [Government department] must listen to critics.*

## Discussion

Care.data was a popular topic of discussion on Twitter following the start of the public information campaign, and the announcement of the delay to the project. Contributors to the Twitter discourse represented a range of interested parties including organisations and individuals directly involved in the care.data project, medical bodies and charities, healthcare providers and researchers, journalists, IT security experts, and patients. Many of the opinions expressed neither conclusively supported nor rejected the idea of a single database of patient information but there was a clear consensus that care.data was a flawed project, in terms of scope, delivery, privacy, security, transparency and management. Some did see the benefits of such a project, which led to the development of the theme ‘potential of care.data and the ideal model of implementation’. Whilst this theme was distinct due to the focus on the positives of care.data, it was also overarching as the ideal model put forward by contributors aimed to address all of the concerns raised to some degree.

**Fig. 2:**
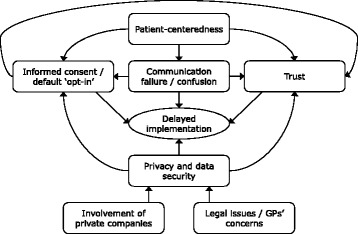
Conceptual map of key themes from Twitter discourse about care.data

The concerns of Twitter users were all interlinked and we have attempted to show how in a conceptual model, which has the delayed implementation of the project at its heart (see Fig. 2). Contributor perceptions of privacy and data security were influenced by their views on the involvement of private companies in terms of the provision of the technology and resources to support the project or their ability to access the records in the database. In addition to these concerns, some felt it would only be acceptable to collect and share fully anonymised data because of the potential to re-identify individuals and risk their privacy, especially as patients had no control over who their data would be available to during the process of data collection and management or afterwards, as they could only stay in or entirely opt-out of the project. The legality of the project was also questioned in relation to the Data Protection Act and GPs had concerns about the availability of the technology required to pseudo-anonymise data before it leaves the GP practice, and the processes that would be put in place to ensure this data was securely transferred and stored.

At the other end of the spectrum, the lack of patient-centeredness was criticised. Patients lack access to their own medical data but are expected to share this with unknown others despite not having been able to contribute to the design of the project. Those involved in the development of care.data were seen as arrogant dictators, telling the population it was in their best interests without explaining why. Many contributors suggested problems could have been avoided, had the project been more patient-centred and involved patients and other stakeholders, such as GPs and data security experts. Thus, this issue influenced trust and opinions about the quality of communication and the default ‘opt-in’.

The failure of the publicity campaign to reach and adequately inform the public was a key issue of concern. The boundaries of the project did not appear to have been specified, as people queried the intended purpose of the database and who the collected data would be made available to. The confusion about care.data and the lack of available information only served to increase distrust and the sense that people were unable to provide informed consent. There was also suggestion that the dissemination method chosen (a leaflet) was decided on the basis of cost, rather than its potential efficacy. The uproar about the leaflet mailout and how few people had read or even received it was seen as the primary reason for the delayed implementation.

Concerns about a ‘junk mail’ approach coupled with the lack of a broader publicity campaign left some feeling there had been a lack of transparency about the project. Some questioned the reasons for this and suspected that information was being withheld. Combined with knowledge of previous failures by the organisations involved in the project, these concerns led to an increased lack of trust and resulted in people opting out and encouraging others to do the same. These queries added to concerns regarding the ethics of making a project such as this opt-out as opposed to opt-in.

It was a combination of all these factors that led to the delay “to allow more time to build understanding of the benefits of using the information, what safeguards are in place, and how people can opt out if they choose to” [11]. However, postponing the project until information about it could be better communicated was not seen as sufficient. Contributors to this discourse called for fundamental changes to be made to the way the project would be delivered. They also specified that implementation should be postponed until all relevant stakeholders (especially patients) had been consulted and there were clear statements regarding how the data would be handled or accessed, by whom, and how security would be maintained. The issue of informed consent also needed to be addressed and the process for opting out, or in, revised. Only by acknowledging people’s concerns and communicating clearly would trust be developed and such a project be seen as acceptable.

Social media sites give individuals and organisations the ability to share information and express their opinions. Twitter enables people from many different backgrounds and perspectives to contribute to a discussion, reviewing the evidence for and against the standpoints and beliefs of others, and provide feedback. Through Twitter, contributors have the potential to educate and persuade those they would not otherwise have been able to communicate with. Some of those engaged in the care.data conversation on Twitter believed that the public had brought about the delay to care.data by being able to voice their opinion through such means, although it is not clear if the decision to delay the project was influenced by this discourse. Twitter is a forum where activists can communicate directly with project leaders and decision makers, or with influential parties such as journalists or advocacy groups but their voices may not be heard or listened to by the relevant parties.

We were unable to find a reliable source of information regarding the demographics of Twitter users but the majority of those contributing to the care.data discussion appeared to be of working-age. Thus we may not have captured the opinions of younger and older, and potentially more vulnerable, populations. A wider range of opinions could have been identified had we captured data from other social media sites, such as Facebook. We focused on Twitter because public tweets containing hashtags can easily be searched and viewed by anyone with access to the internet, regardless of whether or not they have a Twitter account. Furthermore, people use Twitter as a news source, a way of keeping up-to-date with current events, and to communicate with those they don’t necessarily know [13, 14]. However, only those who are aware of care.data could contribute to the discussion, and a survey carried out in June 2014 suggested that over 60 % of the public were still unaware of what care.data was [29].

We may have missed many tweets about the care.data project by only searching for those that included the #caredata hashtag. Furthermore, whilst the NCapture tool for NVivo 10 enabled us to quickly and easily produce comprehensive datasets of tweets, we did not manage to capture a continuous stream. The gaps in data collection could have been avoided had we captured information more frequently. Other tools, such as Spark Streaming or Tweepy, use real-time or ‘live’ data to produce batches of tweets without gaps but these require more technical knowledge and are more resource intensive.

Unlike previous healthcare research studies that have used tweets as a data source, we coded all tweets in our dataset and only used qualitative methods of analysis [19]. This method enabled us to present a more detailed and meaningful analysis than could be derived from counts of the nature of people’s opinions (e.g. sentiment analysis) or through the use of coding algorithms. Counting is a controversial issue in qualitative research and whilst we have attempted to highlight areas of consensus and disagreement we chose to avoid counting the number of tweets per theme or the proportion of contributors that held certain views. This type of ‘credentialing’ counting is used to show the representativeness of data, and requires the objective categorisation of data into themes, which may lead researchers to miss subtle differences. Furthermore, this type of data can introduce bias, as it could be inferred that the most frequently mentioned themes are the most important or significant [30]. The approach we took enabled us to explore the content of tweets in-depth and identify nuances in the contributors’ viewpoints, which would not have been possible had we set out to provide count data as this would have required us to categorise tweets into dichotomies of those that expressed concern about or were accepting of certain issues.

The consensus on Twitter appeared to be that the care.data project is flawed and, as a minimum, requires revision. Those with strong opinions may be more likely to express them through social media and the Twitter discourse cannot be assumed to reflect public opinion especially as Twitter may be used “as a meeting place in which those on one side of the argument … come together to share information and refine and reinforce their own views.” [18]. The findings of a study of public opinion regarding the passing of the Health and Social Care Bill in England supports this suggestion. The authors coded tweets as negative, neutral or supportive and compared their findings to the results of national polling data. The results showed a similar percentage of negative feelings towards the reform but reported that expressions of support were relatively rare on Twitter [17].

Our findings, and those of future studies taking a qualitative approach to analysing tweets, could be strengthened in a number of ways. Supplementary data could be collected from other sources, such as comments on Facebook or “below the line” in online news stories. The authors of posts could be contacted to, with their consent, obtain demographic and background information that would enable data analysis to be done in context. Alternatively, a mixed-methods approach could be taken that would complement the in-depth qualitative analysis with a quantitative analysis to provide a more representative ‘big picture’ view.

## Conclusions

We captured tweets at a time of heated debate about care.data, and when concerns were being raised about the security of healthcare data in general. The use of the #caredata hashtag on Twitter decreased since tweets were captured for this study, for example, only 2,667 such tweets (including retweets) were posted during June and July 2014 [31]. Many of these tweets (455) were posted on 1 July 2014 when the Health Committee inquiry ‘Handling NHS patient data’ received further evidence, having evolved from a more specific enquiry regarding the care.data project.

The ideal model put forward by the contributors to the Twitter discourse featured suggested revisions that addressed all the key areas of concern raised about care.data through this forum. The concerns and recommendations outlined in this paper could be used to modify the project, promote the benefits of ‘big data’ in relation to health, and build trust among the general public. Twitter users expressed the opinion that care.data would not succeed if it went ahead in its current form because of the number of people who would opt-out, the lack of adequate (technological) resources, and the potential for data to fall into the “wrong hands” and be used for non-health related purposes.

If the care.data project were to go ahead without change there could be implications for patient safety in primary care. Not least because a lack of trust in the healthcare service could prevent people from seeking the help they require, leading to under or untimely attendance [32]. A lack of trust could also be detrimental to the patient-provider relationship and lead to communication breakdowns, with patients being taciturn or reticent to disclose certain information [32, 33].

## Abbreviations

DPA: Data Protection Act
GP: General Practitioner
HES: Hospital Episode Statistics
HSCIC: Health and Social Care Information Centre
MP: Member of Parliament
NDPB: Non-Departmental Public Body
SCR: Summary Care Record
